# MiRNA-575 suppresses angiogenesis by targeting Rab5-MEK-ERK pathway in endothelial cells

**DOI:** 10.1042/BSR20181218

**Published:** 2019-01-08

**Authors:** Xin Zhao, Yaping Yi, Chao Meng, Ningyuan Fang

**Affiliations:** Department of Geriatrics, Renji Hospital, School of Medicine, Shanghai Jiaotong University, No. 160 Pujian Road, Pudong New Area, Shanghai 200127, China

**Keywords:** angiogenesis, essential hypertension and atherosclerosis, endothelial cell, miR-575, Rab5

## Abstract

Hypertension is a major risk factor for the development of atherosclerosis. Increased carotid intima-media thickness (CIMT) is generally considered as an early marker of atherosclerosis. Recently, circulating miRNAs have been implicated both as sensitive biomarkers and key regulators in the development of atherosclerosis. However, the biological functions and molecular regulatory mechanisms for miR-575 on angiogenesis remain unknown. In our study, we first identified up-regulation of circulating miR-575 in plasma of essential hypertensive patients with increased CIMT (iCIMT) compared with those patients with normal CIMT (nCIMT). Furthermore, the overexpression of miR-575 in human umbilical vein endothelial cells (HUVECs) by its mimics significantly inhibited migration and proliferation as well as induction of apoptosis of HUVECs. Inhibition of miR-575 performed the reverse effects of HUVECs. We further suggested Rab5B was the downstream target of miR-575 and knockdown of Rab5B significantly inhibited migration and proliferation of HUVECs. Overexpression of Rab5B largely rescued the miR-575-mediated impairment of angiogenesis processes including: cell proliferation, migration, and apoptosis as well as activation of mitogen-activated protein kinase/extracellular signal-regulated kinase (MEK-ERK) signaling. Therefore, our results uncover a novel role of miR-575 in endothelial cells, implying a potential biomarker and clinical target for atherosclerosis in hypertensive patients.

## Introduction

Hypertension has become a major public health issue due to its high global prevalence for decades [1]. Hypertension can lead to target-organ damage such as stroke, heart failure, chronic kidney disease, coronary heart disease, and death [2]. As one of many common causes of target-organ damage in hypertension, atherosclerosis has been known as a predictor of cardiovascular and cerebrovascular diseases [3]. Carotid intima-media thickness (CIMT) measurements are often used to detect early atherosclerosis and assess the risk of cardiovascular diseases. Although accumulated evidences have shown that endothelial dysfunction plays an important part in the progression of atherosclerosis caused by high blood pressure [4], precise molecular mechanisms that attribute to the development of atherosclerosis remain largely unknown.

As a class of highly conserved small endogenous non-coding RNA molecules, miRNAs have been implicated in various aspects of cellular processes including differentiation, proliferation, and apoptosis of cells through post-transcriptional regulation of specific target mRNAs [5]. It has been reported that several miRNAs are directly linked to the progression of inflammation or oxidative stress-induced endothelial cell injury, suggesting importance of miRNAs in the development cardiovascular diseases [6]. Recently, circulating miRNAs have emerged as the key regulator and potential biomarker in cardiovascular diseases [7]. Although numerous studies have shown abnormal expression of miRNAs in plasma of hypertensive patients [8], the roles and mechanisms of circulating miRNAs in the hypertensive patients with target-organ damage such as atherosclerosis are not well explored.

In our study, we identified for the first time that miR-575 was up-regulated in the plasma of essential hypertensive patients with increased carotid intima-media thickness (iCIMT). We further demonstrated the crucial effects of miR-575 on migration, proliferation, and apoptosis of endothelial cells through Rab5B-mediated MAPK signaling pathway, providing the potential role of miR-575 as a biomarker or therapeutic target for atherosclerosis in hypertensive patients.

## Materials and methods

### Clinic specimens

The present study was conducted in accordance with the Declaration of Helsinki. The protocol was approved by the Medical Ethics and Health Committee (permission number: 2017076) in Renji Hospital of Shanghai Jiaotong University. All essential hypertension participants from the department of Geriatrics of Renji Hospital underwent physical examination, ambulatory blood pressure monitoring, and CIMT measurement. A 24-h mean ambulatory BP >130/80 mm Hg was regarded as hypertension [9]. A CIMT value >0.9 mm was defined as vascular damage based on the 2007 Guidelines on the Management of Hypertension [10]. Plasma samples were centrifuged from whole blood at 5000 ***g*** for 10 min at room temperature. Plasma samples were stored at −80 °C until further analysis.

### Plasmids and reagents

The 3′UTR of Rab5B mRNA containing the putative binding site of miR-575 was amplified using cDNA from human umbilical vein endothelial cells (HUVECs) and was cloned into the luciferase reporter psiCHECK-2 plasmid (Promega, Madison City, WI). The human Rab5B coding region was cloned into pcDNA 3.1 to produce pcDNA-Rab5B. The mutant 3′UTR of Rab5B with the seed region for the luciferase reporter was obtained using a KOD Site-Mutagenesis Kit (Toyobo, Japan). MiR-575 mimics and an inhibitor as well as siRNA Rab5B were purchased from Guangzhou RiboBio Co. Ltd., China. The sequences of siRNA, miRNA inhibitor, and mimics were shown in Table 2.

### Cell culture and transfection

HUVECs were obtained from American Type Culture Collection and cultured in Minimum Essential Medium (Invitrogen, Carlsbad, CA) containing 10% FBS (Invitrogen), 100 U/ml penicillin and 0.1 mg/ml streptomycin. HUVECs at passages 6 or 7 were used for all experiments. Plasmid transfection was performed using Lipofectamine 2000 according to the manufacturer’s protocol (Invitrogen). SiRNA, a miR-575 inhibitor, and mimics were transfected using RNAiMAX according to the manufacturer’s instructions (Invitrogen).

### Dual-luciferase reporter assay

HUVECs were plated into 24-well plates and cotransfected with the luciferase reporter and negative control or miR-575 mimics (100 nM). The cells were harvested 24 h after transfection. The luciferase activities of firefly and renilla were detected using the Dual-Luciferase Reporter Assay System (Promega, Madison City, WI) and read on an Envision Luminometer (PerkinElmer, Inc., Waltham, MA).

### Cell proliferation recorded by an MTT assay

HUVECs were plated in a 24-well plate at a density of 1 × 10^4^ cells/well transfected with plasmids, siRNA, and miRNA inhibitor or mimics for the time indicated. Then, a MTT (4, 5-dimethyl-thiazol-2yl)-2, 5-diphe-nyltetrazolium bromide) assay was performed by incubated cells with 0.5 mg/ml MTT for 2 h at 37°C. After incubation, the culture media were removed, and formazan crystals were dissolved by DMSO. The absorbance was measured at 570 nm in a Multiwell Spectrophotometer Plate Reader (BioTek instruments Inc., Winooski, VT).

### Cell proliferation recorded by EdU cell proliferation assay

For EdU proliferation assay, we used Click-iT EdU Alexa Fluor 555 Imaging Kits (Thermo Fisher Scientific, Inc., Waltham, MA) to measure cell proliferation. According to the manufacturer’s instructions, HUVECs were labeled with 10 µM EdU for 30 min and then fixed in 4% paraformaldehyde for 15 min as well as subsequent cell permeabilization with 0.5% Triton X-100. For EdU detection, the cells were stained with Click-iT reaction cocktail for 30 min, followed by staining with DAPI for 30 min in room temperature. At least five different viewing fields were counted for analysis. All images were taken with a Leica SP5 Confocal Microscope (Leica, Germany).

### Wound healing assay

As previously described [11], HUVECs were plated in a six-well plate at 90% confluence after transfection with siRNA, miRNA, or plasmids. A 1 ml pipette tip was used to create a wound scratch relative to the reference point. After washing the cells with the desired media, the first phase-contrast image was acquired based on the reference point by Olympus IX71 Inverted Microscope (Olympus, Japan). After 24 h, a second image was acquired according to the photographed region. The area of wounded region without cell was measured by Image J software.

### Microfluidic cell invasion assay

As previously described [12], HUVECs were plated into the left side of the microfluidic chamber at a density of 1 × 10^5^ cells/well. After transfection with siRNA, miRNA, or plasmids for 24 h, phase-contrast image was captured after invasion for 24 h by Olympus IX71 Inverted Microscope (Olympus, Japan). Invading cell numbers and migrating distance were measured by Image-Pro Plus 5.1 Image Analysis Software.

### Tube formation assay

As previously described, transfected or drug-treated HUVECs were plated in µ-Slide Angiogenesis ibiTreat Microscopy Chamber (Ibidi, Martinsried, Germany) precoated with BD Matrigel Matrix (BD Biosciences, Bedford, MA) for 24 h. Images were captured by Olympus IX71 Inverted Microscope (Olympus, Japan) and total tube lengths were analyzed by Image J Angiogenesis Analyzer (NIH).

### Hoechst 33342 staining for cell apoptosis assay

As previous described [6], we used Hoechst 33342 staining for detecting apoptotic HUVECs. Briefly, HUVECs were plated in 24-well plate at density of 1 × 10^4^ cells/well. After being transfected with miRNA mimics and plasmids with indicated time, cells were stained with Hoechst 33342 for 10 min at 37°C. Then the fluorescent images were captured by Olympus IX71 Inverted Fluorescent Microscope (Olympus, Japan). The apoptotic cell number was quantified with Image-Pro Plus 5.1 Image Analysis Software.

### RNA extraction and real-time quantitative PCR

The total RNAs from human plasma and HUVECs lysates were isolated by using TRIzol reagent according to the manufacturer’s protocol (Invitrogen). For miRNA detection in human plasma, as previously described [13], synthetic *cel-*miR-39 (20 nM) was added in the sample as reference miRNA. miRNA cDNA was reverse-transcribed with a miRNA-specific stem-loop primer by Primescript RT Reagents Kit (Takara, Japan). For mRNA detection, the first-strand cDNA was reverse-transcribed using Superscript III Reverse Transcriptase (Invitrogen). The sequences of primers for miRNAs and mRNAs were shown in Table 3. The qRT-PCR was performed on an ABI PRISM 7500 Sequence Detection System (Applied Biosystems, Foster City, CA) using SYBR Green Master Mix. The qRT-PCR results were analysed using a ΔΔ*C*_t_ method normalized to GAPDH (mRNA) or normalized to *cel*-miR-39 or U6 (miRNA), which is presented as a fold-change.

### Western blotting

HUVECs were lysed in ice-cold lysis buffer (10 mM Tris-HCl, pH 7.4, 150 mM NaCl, 1 mM EDTA, 1% Triton X-100). The lysate was incubated in SDS–PAGE loading buffer for 5 min at 95°C. The samples were separated on an SDS–PAGE gel and probed with primary antibodies (anti-Rab5B, 1:1000, Abcam; anti-cleaved Caspase 3, 1:1000, CST; anti-Caspase 3, 1:1000, CST; anti-phospho mitogen-activated protein kinase (anti-phospho-MEK) (S222), 1:1000, CST; anti-MEK, 1:1000, CST; anti-phospho extracellular signal-regulated kinase (anti-phospho-ERK) (E-4), 1:2000, Santa Cruz; anti-ERK1, 1:2000, Santa Cruz; anti-tubulin, 1:5000, Sigma) and visualized with enhanced chemiluminescent (ECL) HRP substrates (Amersham Biosciences, Litter Chalfont, U.K.). The intensity of bands was quantified with Image-Pro Plus 5.1 Image Analysis Software.

### Statistical analysis

All data are presented as the mean ± S.E.M. The statistical analysis was performed using PRISM (GraphPad Software) with a two-tailed unpaired Student’s *t* test. Differences were considered significant at *P*<0.05.

## Results

### Circulating miR-575 is unregulated in hypertensive patients with iCIMT

**Figure 1:**
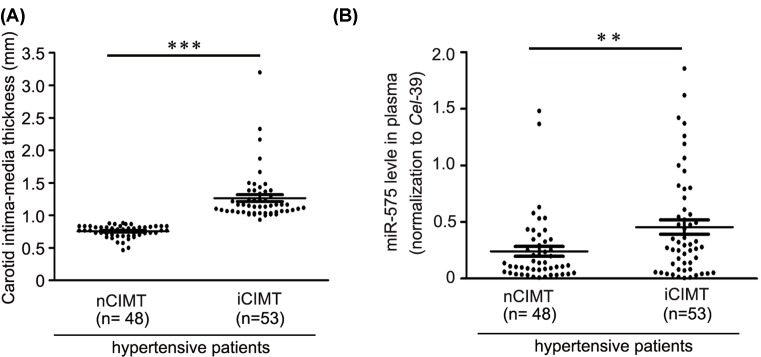
Circulating miR-575 is increased in hypertension patients with iCIMT (**A**) CIMT are measured in hypertensive patients with nCIMT(<0.9 mm, *n*=48) and iCIMT (>0.9 mm, *n*=53). ^***^P<0.001 versus nCIMT. (**B**) A qRT-PCR analysis showed that expression of miR-575 is up-regulated in plasma of essential hypertensive patients with iCIMT (*n*=53). **P<0.01 versus nCIMT.

To investigate the potential function of miR-575 in atherosclerosis, we collected 101 cases of patients with essential hypertension. Based on the measurements of CIMT, control group includes 48 patients with normal CIMT (nCIMT, CIMT <0.9 mm), and the atherosclerosis group includes 53 patients with increased CIMT (iCIMT, CIMT >0.9 mm) (Figure 1A). The demographic, clinic, and laboratory characteristics among groups were summarized in Table 1. There were no significant differences in age, total cholesterol (TC), triglyceride (TG), high-density lipoprotein cholesterol (HDL-C), low density lipoprotein cholesterol (LDL-C), and fasting blood-glucose (FBG) (Table 1). Furthermore, we found that expression of circulating miR-575 was significantly up-regulated in plasma of essential hypertensive patients with iCIMT compared with those nCIMT patients (Figure 1B), indicating the potential function of miR-575 in the progression of atherosclerosis.

**Table 1:** Demographic, clinic, and laboratory characteristics among groups

	Hypertension with nCIMT (*n*=48)	Hypertension with iCIMT (*n*=53)	*P*-value
Age (years)	72.06 ± 6.85	72.45 ± 6.39	0.768
TC (mmol/l)	3.83 ± 0.59	3.71 ± 0.62	0.331
TG (mmol/l)	0.95 ± 0.33	0.86 ± 0.34	0.195
HDL-C (mmol/l)	1.22 ± 0.28	1.21 ± 0.29	0.890
LDL-C (mmol/l)	2.16 ± 0.43	2.01 ± 0.54	0.128
FBG (mmol/l)	4.69 ± 0.54	4.71 ± 0.53	0.842
ALT (U/l)	20.19 ± 10.78	18.89 ± 11.32	0.556
AST (U/l)	24.31 ± 6.94	22.81 ± 6.66	0.271
BUN (mmol/l)	5.84 ± 1.31	5.50 ± 1.40	0.208
Scr (umol/l)	71.42 ± 18.52	71.18 ± 18.81	0.948
UA (umol/l)	291.04 ± 72.73	275.43 ± 69.99	0.276
24 h mean SBP (mmHg)	136.24 ± 9.50	145.45 ± 7.60	0.000^***^
24 h mean DBP (mmHg)	79.49 ± 8.30	83.79 ± 8.73	0.013^*^
24 h daytime SBP (mmHg)	137.79 ± 10.07	146.82 ± 8.32	0.000^***^
24 h daytime DBP (mmHg)	80.68 ± 9.436	85.01 ± 9.61	0.025^*^
24 h nighttime SBP (mmHg)	129.32 ± 19.04	140.34 ± 18.73	0.004^**^
24 h nighttime DBP (mmHg)	74.25 ± 10.54	79.69 ± 10.93	0.013^*^
CIMT (mm)	0.72 ± 0.014	1.26 ± 0.055	0.000^***^
MiRNA-575 expression level	0.241 ± 0.043	0.456 ± 0.063	0.007^**^

Abbreviations: ALT, alanine transaminase; AST, aspartate transaminase; BUN, blood urea nitrogen; DBP, diastolic blood pressure; iCIMT, increased carotid intima-media thickness; nCIMT, normal carotid intima-media thickness; SBP, systolic blood pressure; Scr, serum creatinine; UA, uric acid.

^*^P<0.05, ^**^P<0.01, and ^***^P<0.001.

**Table 2:** The sequences of miRNAs and siRNAs used in the present study

miRNA/siRNA/shRNA	Sequence (5′−3′)
miR-575 mimics	GAGCCAGUUGGACAGGAGC
miR-575 inhibitor	GCUCCUGTCCAACUGGCUC
Negative control	UUUGUACUACACAAAAGUACUG
si-Rab5B	CCUUAAUGUAAAUGAACUGG
si-control	ACGTGACACGTTCGGAGAATT

**Table 3:** The sequences of primer for qRT-PCR in the present study

Gene/miRNA	Forward primer (5′−3′)	Reverse primer/reverse transcription primer (5′−3′)
*Human Rab5B*	AAGGAACTACAGCGACAGGC	GCTCTTGTTCTGCTGGGACT
*Human Gapdh*	GCACCGTCAAGGCTGAGAAC	TGGTGAAGACGCAGTGGA
miR-575	ACACTCCAGCTGGGGAGCCAGTTGGACAGG	TCAACTGGTGTCGTGGAGTCGGCAATTCAGTTGAGGCTCCTGT
U6	ACACTCCAGCTGGGCGCAAATTCGTGAAGC	CTCAACTGGTGTCGTGGAGTCGGCAATTCAGTTGAGAAAAATAT
Universal reverse primer		CTCAACTGGTGTCGTGGAGTCGG

### MiR-575 regulates migration and proliferation of endothelial cell

**Figure 2:**
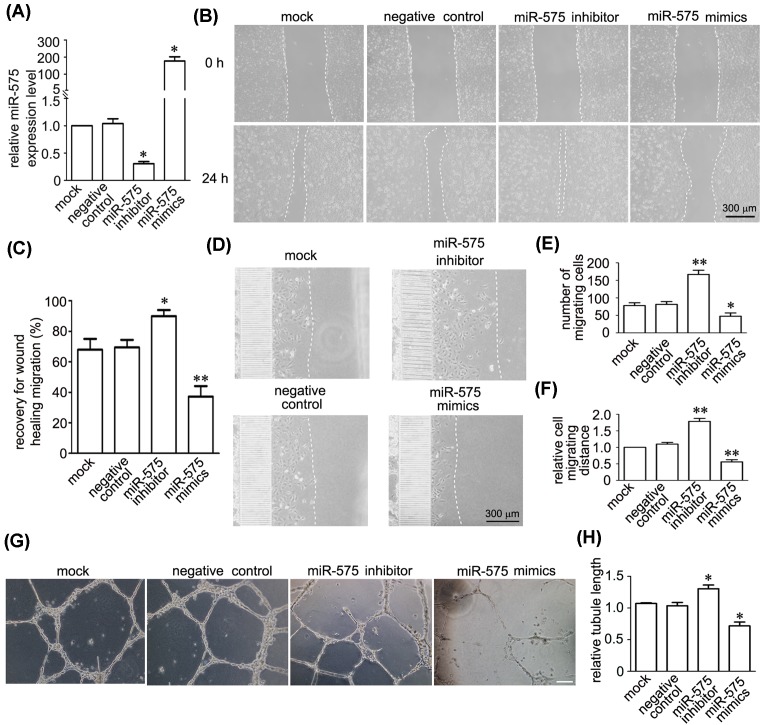
MiR-575 inhibits migration, proliferation, and tube formation of endothelial cells (**A**) A qRT-PCR analysis showing that miR-575 was up-regulated by miR-575 mimics and down-regulated by a miR-575 inhibitor in HUVECs. *P<0.05, **P<0.01 versus the negative control (*n*=3). (**B**,**C**) Representative images (**B**) and quantitative data (**C**) showed that miR-575 regulates migration of endothelial cells by wound healing assay. *P<0.05, **P<0.01 versus the negative control (*n*=4). Scale bar, 300 µm. (**D–F**) Representative images (**D**) and quantitative data (**E**,**F**) showed that miR-575 regulates migrated number of endothelial cells and distance by microfluidic cell invasion assay. *P<0.05, **P<0.01 versus the negative control (*n*=3). Scale bar, 300 µm. (**G**,**H**) Representative images (**G**) and quantitative data (**H**) showed that the inhibition of miR-575 increased total tube length whereas overexpression of miR-575 decreased total tube length in cultured HUVECs after post-plating for 24 h. The results are presented as the mean ± SEM. *P<0.05 versus negative control (*n*=3). Scale bar, 100 µm.

To determine the possible roles of miR-575 in endothelial cells, we first successfully used miR-575 inhibitor and its mimics for down-regulation and up-regulation of miR-575 in HUVECs (Figure 2A). Then we examined the cell migration by wound healing experiment. The result showed that inhibition of miR-575 significantly accelerated cell migration, and overexpression of miR-575 largely decreased the migration of endothelial cells (Figure 2B,C). Meanwhile, we evaluated cell migration using a microfluidic cell invasion assay. After 24 h culture in one side of microfluidic chamber, results showed that applying miR-575 inhibitor increased the migrated cell numbers and migrating distances, whereas miR-575 mimics performed the opposite effects, suggesting the critical function of miR-575 on endothelial cell migration (Figure 2D–F). To determine the effect of miR-575 in angiogenesis, we performed tube formation assay to test effects of miR-575 in VEGF-induced endothelial angiogenesis. Results showed that knockdown of miR-575 increased total tube length after VEGF treatment, whereas overexperssion of miR-575 impaired tube formation, indicating negative impact of miR-575 on VEGF-induced angiogenesis (Figure 2G,H).

### MiR-575 regulates apoptosis of endothelial cell

**Figure 3:**
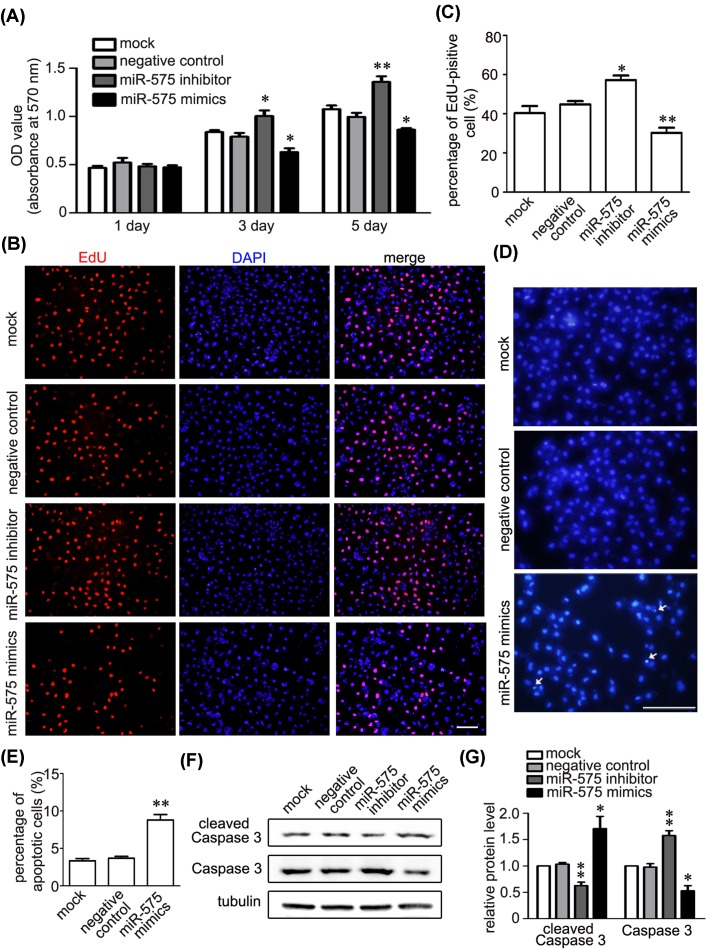
miR-575 positively promotes apoptosis of endothelial cells (**A**) MTT assays showing that applying a miR-575 inhibitor or its mimics increased or decreased proliferation of HUVECs at day 3 and 5. *P<0.05, **P<0.01 versus the negative control (*n*=4). (**B**,**C**) Representative EdU images (**B**) and quantitative data (**C**) showing that HUVECs cell proliferation was increased by inhibition of miR-575 and decreased by miR-575 mimics. The results are presented as the mean ± S.E.M. ^*^P<0.05 and ^#^*P*<0.05 versus the indicated treatments (*n*=3). Scale bar, 200 µm. (**D**,**E**) Representative images of Hoechst 33342 staining (**D**) and quantitative data (**E**) showed that miR-575 mimics induced apoptosis of HUVECs. ^**^P<0.01 versus the negative control (*n*=3). Scale bar, 200 µm. (**F**,**G**) Representative immunoblots (**F**) and quantitative data (**G**) showed that miR-575 mimics increased the protein level of cleaved Caspase 3. *P<0.05, **P<0.01 versus the negative control (*n*=3).

We then further assessed the function of miR-575 in the proliferation of HUVECs by MTT and EdU assays. Down-regulation of miR-575 by its inhibitor significantly increased cell proliferation at day 3 and 5 (Figure 3A–C), whereas overexpression of miR-575 decreased cell proliferation, indicating the importance of miR-575 in the viability of endothelial cells. Moreover, we determined the effects of miR-575 on cell apoptosis using Hoechst 33342 staining. The result showed that miR-575 mimics significantly promoted the apoptotic cell death of HUVECs (Figure 3D,E). Meanwhile, cleaved Caspase3 was elevated in overexpression of miR-575 on HUVECs, whereas total Caspase3 were down-regulated (Figure 3F,G), further suggesting the importance of miR-575 in the cell viability of HUVECs.

### Rab5B is the downstream target for miR-575

**Figure 4:**
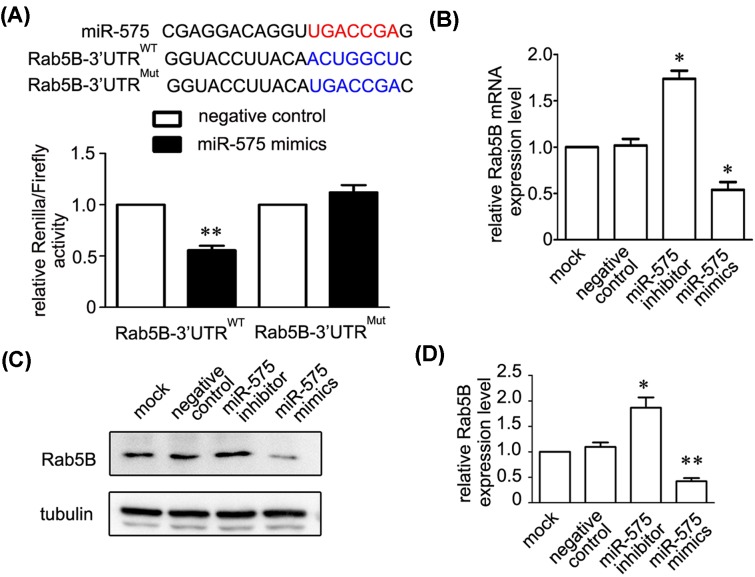
Rab5B is the downstream target of miR-575 (**A**) Luciferase reporter assays showed that Rab5B is a potential downstream target of miR-575. The miR-575 seed sequence is highlighted in red, and the seed match sequences of 3′UTR for *Rab5B* are highlighted in blue (upper panel). The luciferase activity of the 3′UTR^WT^, but not the mutated Rab5B, was down-regulated by miR-575 mimics in HUVECs (down panel). ^***^P<0.001 versus the negative control (*n*=3). (**B**–**D**) qRT-PCR (**B**) and immunoblots (**C**,**D**) showed that applying miR-575 inhibitor or mimics increased and decreased the mRNA and protein level of Rab5B. Tubulin was used as a loading control. ^*^P<0.05, ^**^P<0.01 versus the negative control (*n*=3).

To identify the possible downstream target of miR-575 in HUVECs, we used miRNA targets predication tools including miRanda and TargetScan. We found a miR-575 punitive interaction site within the 3′UTR of Rab5B (Figure 4A). A lucriferase reporter experiment showed that miR-575 mimics dramatically decreased 3′UTR activity of Rab5B but not of its mutant form, which lost the ability to be targeted with miR-575 (Figure 3A). Furthermore, we assessed the mRNA and protein level of Rab5B after knockdown and overexpression of miR-575. The qRT-PCR and immunoblotting results showed that down-regulation or up-regulation of miR-575 bidirectionally regulated mRNA and protein level of Rab5B (Figure 4B–D).

### Rab5B regulates proliferation and migration of endothelial cells

**Figure 5:**
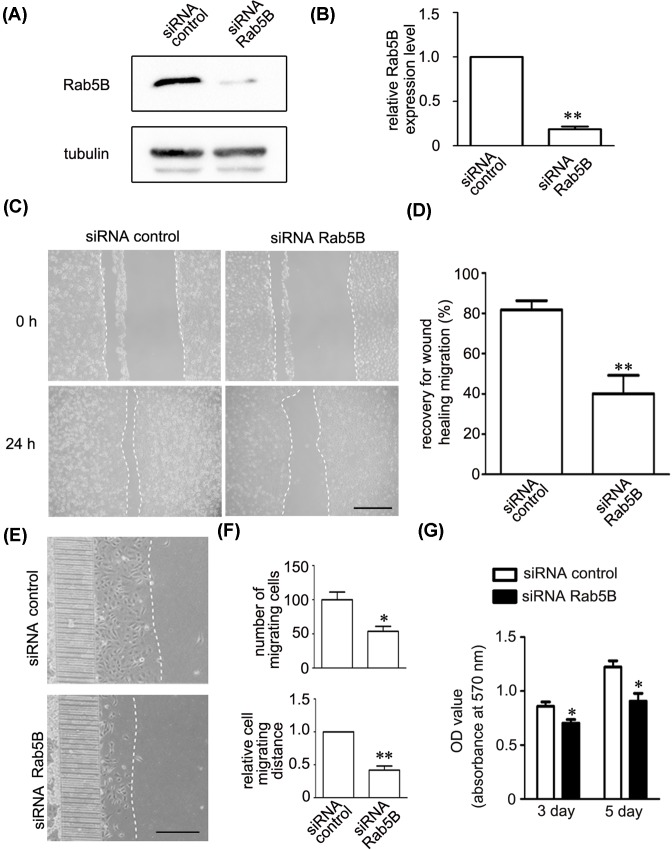
Rab5B regulates migration and proliferation of endothelial cells (**A**,**B**) Representative immunoblots (**A**) and quantitative data (**B**) showing that applying siRNA Rab5B dramatically decreased the protein level of Rab5B. Tubulin was used as a loading control. ^**^P<0.01 versus the siRNA control (*n*=3). (**C**,**D**) Representative images (**C**) and quantitative data (**D**) showed that applying siRNA Rab5B decreased migration of HUVECs by wound healing assays. ^**^P<0.01 versus the siRNA control (*n*=3). Scale bar, 300 µm. (**E**,**F**) Representative images (**E**) and quantitative data (**F**) showed that applying siRNA Rab5B the decreased migrated number of endothelial cells and distance by microfluidic cell invasion assay. ^*^P<0.05, ^**^P<0.01 versus the siRNA control (*n*=3). Scale bar, 300 µm. (**G**) MTT assays showing that applying siRNA Rab5B decreased the proliferation of HUVECs at day 3 and 5. ^*^P<0.05, versus siRNA control (*n*=4).

We then evaluated the effects of Rab5B on angiogenesis of endothelial cell. Transfection with siRNA Rab5B dramatically reduced the endogenous protein level of Rab5B (Figure 5A,B). Then we assessed the biological functions of Rab5B in cell migration and proliferation. The results of wound healing and microfluidic cell invasion assay showed that the knockdown of Rab5B significantly reduced migration of HUVECs (Figure 5C–F). Moreover, MTT assay showed that knockdown of Rab5B decreased proliferation of HUVECs at day 3 and 5 (Figure 5G).

### The effects of miR-575 are rescued by overexpression of Rab5B

**Figure 6:**
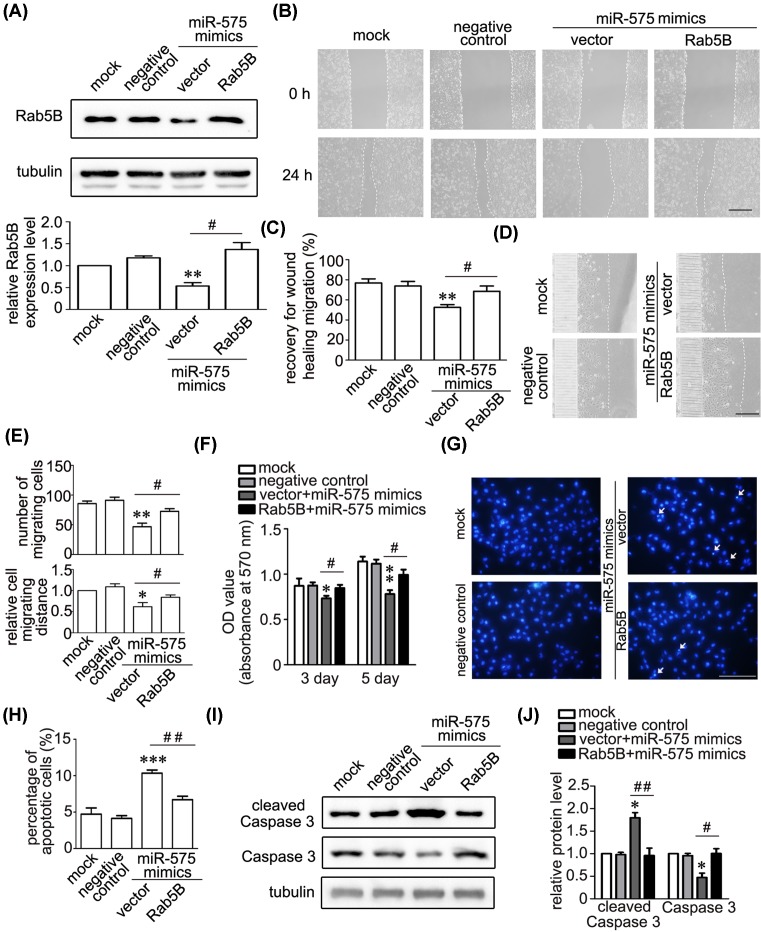
Rab5B rescued the effects of miR-575 on migration, proliferation, and apoptosis of endothelial cells (**A**) Representative immunoblots and quantitative data showing that overexpression of Rab5B rescued miR-575-mediated down-regulation of Rab5B. Tubulin was used as a loading control. ^*^P<0.05 versus negative control, and ^##^*P*<0.01 versus indicated treatments (*n*=3). (**B**,**C**) Representative images (**B**) and quantitative data (**C**) showed that the overexpression of Rab5B rescued miR-575-impaired migration of HUVECs. ^**^P<0.01 versus negative control, and ^#^*P*<0.05 versus indicated treatments (*n*=3). Scale bar, 300 µm. (**D**,**E**) Representative images (**D**) and quantitative data (**E**) showed that the overexpression of Rab5B rescued miR-575-mediated decrease of migrated cell numbers and distance by microfluidic cell invasion assay. ^*^P<0.05, ^**^P<0.01 versus negative control, and ^#^*P*<0.05 versus indicated treatments (*n*=3). Scale bar, 300 µm. (**F**) MTT assays showing that overexpression of Rab5B rescued miR-575-mediated decrease of endothelial cell proliferation at day 3 and 5. ^*^P<0.05, ^**^P<0.01 versus negative control, and ^#^*P*<0.05 versus indicated treatments (*n*=4). (**G**,**H**) Representative images (**G**) of Hoechst 33342 staining and quantitative data (**H**) showed that the overexpression of Rab5B partially rescues miR-575-induced apoptosis of HUVECs. ^***^P<0.001 versus negative control, and ^##^*P*<0.01 versus indicated treatments (n=3). Scale bar, 200 µm. (**I**,**J**) Representative immunoblots (**I**) and quantitative data (**J**) showing that the overexpression of Rab5B rescued miR-575-mediated down-regulation of cleaved Caspase 3. Tubulin was used as a loading control. ^*^P<0.05 versus negative control; and ^#^*P*<0.05, ^##^*P*<0.01 versus indicated treatments (*n*=3).

To further confirm miR-575-mediated specific effects, we constructed coding region of Rab5B to perform the rescue experiment. The immunoblots results showed that miR-575-mediated down-regulation of Rab5B were completely rescued by overexpression of Rab5B (Figure 6A). Then we evaluate the effects of Rab5B in miR-575-impaired cell migration and proliferation as well as miR-575-induced cell apoptosis. The result showed that overexpression of Rab5B significantly rescues miR-575-mediated decrease of cell migration by wound healing and microfluidic cell invasion as well as cell proliferation by MTT assays (Figure 6B–F). Further evidence showed that overexpression of Rab5B largely rescues miR-575-induced cell apoptosis and increased cleaved Caspase 3 (Figure 6G–J). Taken together, our results demonstrated that miR-575-induced suppression on angiogenesis of endothelial cell is mediated largely by Rab5B.

### miR-575 suppresses MEK-ERK signaling by targeting Rab5B

**Figure 7:**
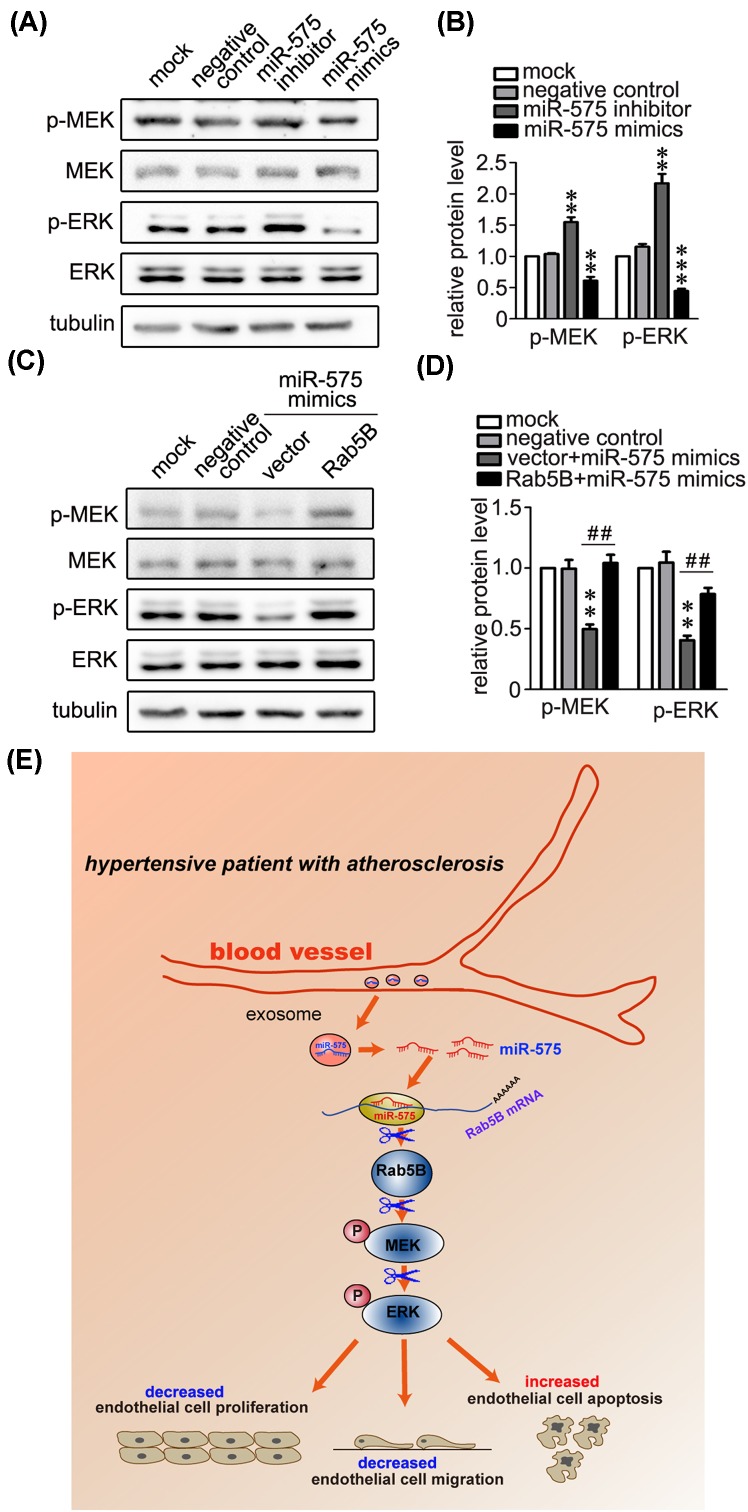
MiR-575 suppresses MEK-ERK signaling by targeting Rab5B (**A**,**B**) Representative immunoblots (**A**) and quantitative data (**B**) showing that applying miR-575 inhibitor or mimics increased and decreased phosphorylated MEK and ERK in HUVECs. ERK, MEK, and Tubulin were used as loading controls. ^*^P<0.05, ^**^P<0.01 versus negative control (*n*=3). (**C**,**D**) Representative immunoblots (**C**) and quantitative data (**D**) showing that the overexpression of Rab5B rescued miR-575-mediated decrease of phosphorylated MEK and ERK. MEK, ERK, and tubulin were used as loading controls. ^**^P<0.01 versus negative control, and ^##^*P*<0.01 versus indicated treatments (*n*=3). (**E**) A proposed model for illustrating the possible role of miR-575 in vascular integrity. Up-regulated plasma level of miR-575 in essential hypertensive patients with atherosclerosis suppresses angiogenesis processes including migration, proliferation, and apoptosis of endothelial cells, via targeting Rab5B and MEK-ERK signaling pathways.

Rab family proteins have been suggested to be involved in distinct signaling pathways [14]. Thus we determined the possible impacts of miR-575 on MAPK signaling pathway. Immunoblots results showed that the inhibition of miR-575 enhanced the phosphorylated MEK and ERK activity, whereas overexpression of miR575 caused an apparent reduction (Figure 7A,B), indicating miR-575 may be involved in Rab5 signaling pathway. Moreover, we further found that miR-575-mediated deactivation of MEK-ERK pathways was also rescued by overexpression of Rab5B, demonstrating the importance of miR-575 in Rab5b-MEK-ERK signaling pathway (Figure 7C,D). Thus, we summarized a model that increased plasma level of miR-575 in essential hypertensive patients with atherosclerosis suppresses angiogenesis processes including migration, proliferation, and apoptosis of endothelial cells via targeting Rab5B and MEK-ERK signaling pathways (Figure 7E).

## Discussion

Essential hypertension is a crucial risk factor for the progression of target-organ damage such as atherosclerosis. The viability and migration of endothelial cells are key components in the progress of cardiovascular diseases. Circulating miRNAs have been recently focused as the potential marker in the progress of cardiovascular diseases [15]. Our study identified that a novel circulating miRNA, miR-575, was up-regulated in the plasma of essential hypertension patients with atherosclerosis. Further evidence showed miR-575 contributes in the proliferation, migration, and apoptosis of endothelial cells by targeting Rab5 and its downstream MEK-ERK signaling pathway.

Essential hypertension is one of the most serious health problems because of its high global prevalence [1]. More recently, there is a mounting evidence that circulating miRNAs have the potential to be a biomarker in target-organ damage such as atherosclerosis [7]. Previous report has identified that atherosclerosis-related circulating miR-21 and miR-361 are sensitive predictors in acute myocardial infarction [16]. In our study, expression level of circulating miR-575 in plasma of essential hypertensive patients with iCIMT is higher than those hypertensive patients with nCIMT, implying the critical function of miR-575 in progression of atherosclerosis. Several dysregulated circulating miRNAs such as miR-210 and miR-425 have been previously found in hypertension patients compared with healthy individuals [7]. However, the circulating biomarker for atherosclerosis in hypertensive patients is not explored. Interestingly, recent report also showed that the expression of circulating let-7 is associated with iCIMT in hypertensive patients [17], indicating distinct circulating miRNA-mediated regulation during the progression of atherosclerosis for hypertensive patients.

The biological functions of miR-575 are rarely explored in endothelial cells during angiogenesis. Our study first investigated the effects of miR-575 on angiogenesis including migration, proliferation, and apoptosis of HUVECs. Several studies have been proved that miR-575 was dysregulated in many tumors such as gastric cancer and non-small cell lung cancer [18,19]. A previous report showed miR-575, as an oncogene, performs positive influence on various aspects of non-small lung cancers including cell proliferation, cell migration and cell invasion [19]. However, our data suggested that miR-575 negatively regulates cell proliferation and migration of endothelial cells. The possible explanation is due to the distinct targets for miR-575 in endothelial cells or in lung cancer cells. MiR-575 induced proliferation of lung cancer cell by down-regulation of BLID, which is a cell death inducer, supporting multiple functions of miR-575 in different cellular contexts. More interestingly, a recent report showed increased expression of miR-575 was involved in placental development by increasing apoptosis of choriocarcinoma cells, which is consistent with miR-575 effects on apoptosis of endothelial cells.

We further identified that Rab5B was the downstream target of miR-575 by luciferase reporter assay. As a small GTPase, Rab5 is participating in various cellular functions including formation, transition, and motility of early endosomes [20]. Our data suggested that down-regulation of Rab5B by siRNA or overexpression of miR-575 inhibit cell migration and proliferation which is consistent with Rab5 activity in the metastatic cancer cells [21,22]. Previous study also showed that the loss of Rab5 could induce cell apoptosis by deactivating ERK signaling [23]. Our result also proves that miR-575 regulates phosphorylated MEK-ERK by targeting Rab5, suggesting the importance of Rab5 on angiogenesis of endothelial cell. Accumulated evidences suggest that activation of MEK-ERK pathway is responsible for cell proliferation and migration as well as cell apoptosis [24]. Our rescue experiment further showed that overexpression of Rab5B partially rescued the miR-575 effects, indicating contributions of other targets involved in the miR-575-mediated inhibition on endothelial angiogenesis.

## Conclusion

In summary, our results showed that plasma level of miR-575 significantly increased in essential hypertensive patients with atherosclerosis. Furthermore, miR-575 suppresses angiogenesis processes including migration and proliferation as well as induction apoptosis of endothelial cells through targeting Rab5B and subsequent MEK-ERK signaling pathway. Our study sheds new light on the circulating miR-575 as a potential biomarker and therapeutic target in the progression of atherosclerosis caused by high blood pressure.

## Abbreviations

CIMT: carotid intima-media thickness
CST: Cell Signaling Technology
EdU: 5-ethynyl-2’-deoxyuridine
ERK: extracellular signal-regulated kinase
GAPDH: glyceraldehyde 3-phosphate dehydrogenase
HRP: Horseradish Peroxidase
HUVEC: human umbilical vein endothelial cell
MEK: mitogen-activated protein kinase
MTT: 4, 5-dimethyl-thiazol-2yl)-2, 5-diphe-nyltetrazolium bromide
qRT-PCR: real-time quantitative reverse transcription
VEGF: Vascular endothelial growth factor
